# Neutrophil to lymphocyte ratio and platelet to lymphocyte ratio as prognostic predictors for delirium in critically ill patients: a systematic review and meta-analysis

**DOI:** 10.1186/s12871-023-01997-2

**Published:** 2023-02-21

**Authors:** Shirin Sarejloo, Niloofar Shojaei, Brandon Lucke-Wold, Rebecca Zelmanovich, Shokoufeh Khanzadeh

**Affiliations:** 1 Cardiovascular Research Center, Shiraz University of Medical Sciences, Shiraz, Iran; 2 School of Medicine, Zanjan University of Medical Sciences, Zanjan, Iran; 3 Department of Neurosurgery, University of Florida, Gainesville, USA; 4 University of Central Florida College of Medicine, Orlando, USA; 5 Student Research Committee, Tabriz University of Medical Sciences, Tabriz, Iran

**Keywords:** Neutrophil to lymphocyte ratio, Platelet to lymphocyte ratio, Delirium, Stroke, COVID-19, Post-operative delirium

## Abstract

**Introduction:**

In this systematic review and meta-analysis, we aim to analyze the current literature to evaluate neutrophil to lymphocyte ratio (NLR) and platelet to lymphocyte ratio (PLR) values among critically ill patients who develop delirium as compared to those who do not.

**Methods:**

PubMed, Web of Science, and Scopus were used to conduct a systematic search for relevant publications published before June 12, 2022. The Newcastle–Ottawa scale was used for quality assessment. Because a significant level of heterogeneity was found, we used the random-effects model to generate pooled effects.

**Results:**

Twenty-four studies including 11,579 critically ill patients, of whom 2439 were diagnosed with delirium, were included in our meta-analysis. Compared with the non-delirious group, the delirious group's NLR levels were significantly higher (WMD = 2.14; CI 95% = 1.48–2.80, *p* < 0.01). In the subgroup analysis according to the type of critical condition, the NLR levels in patients of delirious group were significantly more than those of non-delirious group in studies on POD, PSD and PCD (WMD = 1.14, CI 95% = 0.38–1.91, *p* < 0.01, WMD = 1.38, CI 95% = 1.04–1.72, *p* < 0.001, and WMD = 4.22, CI 95% = 3.47–4.98, *p* < 0.001, respectively). However, compared with the non-delirious group, the delirious group's PLR levels were not significantly different (WMD = 1.74; CI 95% = -12.39–15.86, *p* = 0.80).

**Conclusion:**

Our findings support NLR to be a promising biomarker that can be readily integrated into clinical settings to aid in the prediction and prevention of delirium.

**Supplementary Information:**

The online version contains supplementary material available at 10.1186/s12871-023-01997-2.

## Background

Delirium is a serious neuropsychiatric illness that is characterized by acute fluctuations in mental status associated with altered consciousness, emotional disturbances, and inattention [1]. It is often seen in the setting of acute illness. however, it can also be precipitated by additional physiologic stressors such as medications, electrolyte abnormalities, and dehydration [2].

Once thought to be a transient disorder of insignificant consequence, delirium is now recognized as a serious medical condition with important impacts on morbidity and mortality. The prevalence of delirium in the intensive care unit (ICU) ranges from 32–48% and can increase up to 83% in mechanically ventilated patients [3–6]. Those who develop delirium are at significantly greater risk for persistent cognitive decline, institutionalization, and post-discharge mortality [7, 8].

There are several well-characterized predisposing risk factors for delirium, such as advanced age, pre-existing cognitive impairment, psychiatric illness, and co-morbid disease [1]. These risk factors, when in the presence of a precipitating event such as critical illness, lead to the onset of delirium [1].

Despite well-characterized risk factors, the underlying pathophysiology of disease remains poorly understood. As a result, our ability to treat and prevent disease is limited. Currently, several pathogenic pathways such as neuroinflammation, neuroendocrine dysfunction, oxidative stress, and neurotransmitter imbalance have all been proposed [1, 9]. Despite their inherent differences, each appears to intersect on a final common pathway of impaired neural network connectivity [1, 9].

As it pertains to critically ill patients, neuroinflammation likely holds a central role in pathogenesis [10]. As a result, a number of inflammatory markers have been studied in this context [11]. Despite the promise, results have remained inconclusive, and many of these markers, such as cytokines, remain technically difficult to implement clinically [12].

Thus, attention has turned to more novel markers of inflammation. Neutrophil to lymphocyte ratio (NLR) and platelet to lymphocyte ratio (PLR) are emerging inflammatory markers that have gained increased attention in the setting of delirium. Importantly, NLR and PLR are cheap, available measures obtained from a white blood cell count differential that can be readily adapted into clinical practice. They are established markers of inflammation for a variety of diseases and are well-known for their prognostic utility. Specifically, NLR reflects the dynamic online relationship between neutrophils, the dominant players in innate immunity that serve to amplify pro-inflammatory responses, and lymphocytes, the components of the adaptive immune system which serve to regulate immune responses. It can predict prognosis in a variety of conditions such as cancer, sepsis, COVID-19 infection, cardiovascular disease, and diabetes [13, 14]. Similarly, PLR has been studied as a prognostic tool for inflammatory and vascular conditions, such as Kawasaki’s disease, rheumatoid arthritis, and cardiovascular disease [15, 16]. Given the pathogenic role of systemic inflammation in delirium, both PLR and NLR have potential as prognostic markers in this setting [17–35].

Several recent studies have explored the predictive utility of these measures with promising results [17–35]. However, overall efficacy remains unclear. Therefore, in this systematic review and meta-analysis, we aim to analyze the current literature to evaluate NLR and PLR values among critically ill patients who develop delirium as compared to those who do not. The results of this study can serve to validate NLR and PLR as emerging prognostic markers for delirium while simultaneously elucidating pathophysiology.

## Methods

### Search strategy

As shown in Supplementary Material A this meta-analysis was carried out according to the Preferred Reporting Items for Systematic Review and Meta-analyses statement's(PRISMA) guidelines [36]. PubMed, Web of Science, and Scopus were used to conduct a systematic search for relevant publications published before March 2019, and again up to June 2022 to identify any new studies. The search strategy was as follow: (delirium) AND ( (neutrophil AND lymphocyte AND ratio) OR ( neutrophil-to-lymphocyte) OR NLR) OR ( ( platelet AND lymphocyte AND ratio) OR ( platelet-to-lymphocyte) OR PLR). We provided the specific search strategy for each database in Supplementary Material B. We looked for articles in all languages and had them translated as needed. Articles were also found by manually scanning the references inside identified articles and using PubMed's "related articles" tool. The titles/abstracts of the articles collected were separately investigated by two authors. Then the entire texts of relevant studies were individually reviewed by the same two writers for eligibility. In both stages, a third independent author resolved any differences between authors. Our study was registered with the PROSPERO (CRD42023351185).

### Criteria for inclusion and exclusion

We identified eligible literature based on the PICOS (population, intervention, control, outcomes, and study design) principle to ensure a systematic search. The inclusion criteria were as follows.

- Population: The study population was defined as critically sick individuals with delirium. Critical ill patients were considered patients who experienced major stress such as severe COVID-19 cases, stroke cases, those undergoing surgeries, or any other patients admitted to ICU due to their critical condition.
- Intervention: Researches obtaining NLR or PLR data from critically ill individuals with delirium.
- Control: Critically ill individuals without delirium. Only researches comparing NLR and PLR data from critically sick individuals with delirium against critically ill individuals without delirium were included.
- Outcomes: Diagnostic role of NLR and PLR
- Studies: Cohort, cross-sectional, and case–control studies were included for analysis.

The criteria for exclusion were as follows: 1) researches with similar data; 2) animal studies, letters to editors, reviews, case series, and case reports.

### Data extraction

The first author, year of publication, study design, study location, type of critical illness requiring ICU care (COVID-19 or stroke or major surgery), total sample size, definition of delirium, number of delirious and non-delirious cases, mean and SD of NLR and PLR levels, or any data for estimating the mean and SD (median and IQR or/and range), cut-off value of NLR and its false/true positive and false/true negative from 2 × 2 table were all extracted manually by two author (Sh.Kh. and B.L.). When the number of patients in false/true positive and false/true groups was not reported, we calculated it using sensitivity, and specificity.

### Quality assessment

Two authors assessed the quality of each study using the Newcastle–Ottawa scale (NOS), including three components: selection of the cohort, comparability of cohorts on the basis of the design or analysis, how the exposure was ascertained, and how the outcomes of interest were assessed [37]. Disagreements between the authors were finally resolved through consensus. Studies with scores of six or more were considered to be of high quality.

### Statistical analysis

The meta-analysis of included studies was performed using Stata 11.2 software (Stata Corp, College Station, TX). We used weighted mean difference (WMD) with 95% confidence interval (CI) to assess the differences in the NLR and PLR levels between delirious and non-delirious patients, because studies did used different units for NLR and PLR.

The I ^2^ and Cochran's Q tests were adopted to determine the heterogeneity across the included studies. The I ^2^ > 50% and *p*-value of Q test < 0.05 were conceived as significant heterogeneity between studies. Finally, because a significant level of heterogeneity was found, we used the random-effects model to generate pooled effects. Subgroup analysis was performed to evaluate the effects of study design (retrospective and prospective), type of critical illness (post-operative delirium(POD), post-COVID delirium(PCD), and post-stroke delirium(PSD)), definition of delirium (according to DSM, CAM, DSM + CAM, RAS + CAM, other definitions (ICDSC, 4AT, validate chart-based method), and study location (East Asia, Turkey, Europe, and Americas). Summary receiver operating characteristic (SROC) curve, the sensitivity, specificity, diagnostic odds ratio (DOR), negative likelihood ratio, and positive likelihood ratio were assessed using “metandi” command and used to determine diagnostic value of NLR for delirium. In addition, we used the funnel plot and Egger test to determine the publication bias.

## Results

### Search and selection of literature

**Fig. 1:**
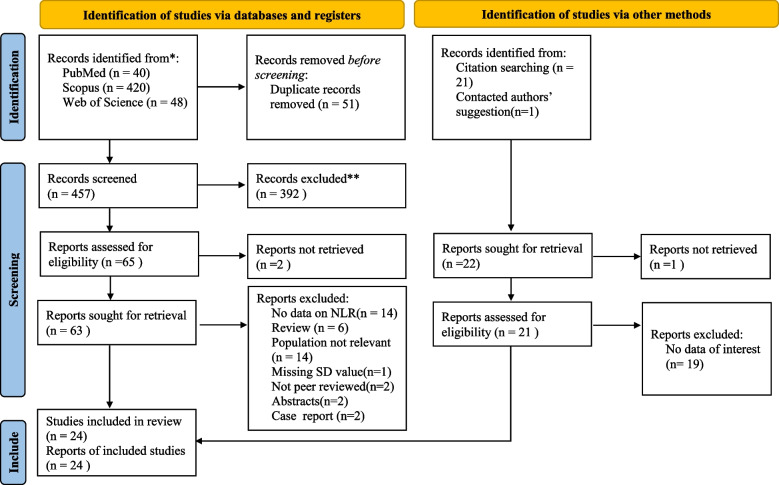
Flow chart of search and study selection

A total of 530 records were retrieved in the database search and manual search of citation list of articles. After the exclusion of duplicates and not relevant records, 24 studies [17–35, 38–42] were included in the qualitative and qualitative analysis. A flow chart depicting the selecting process is shown in Fig. 1.

### Characteristics of the included studies

This meta-analysis included 24 studies [17–35, 38–41, 43], 17 of which were retrospective [18–20, 22, 23, 25, 26, 29–33, 35, 38–41] and seven prospective [17, 21, 24, 27, 28, 34, 43]. In terms of document language, all of the documents were written in English language.

In total, 11,579 critically ill patients were enrolled in the studies, with 2439 developing delirium. Table 1 shows the overall characteristics of the studies and their quality scores. Supplementary Material C presented the methodological quality assessment of all studies. In total, 23 research [17–35, 38–41] examined NLR levels in delirious and non-delirious patients, seven studies [22–24, 29–31, 35, 41] reported PLR levels in delirious patients versus non-delirious patients, and five studies reported diagnostic value of NLR in delirium, based on ROC curve analysis [19, 21, 28, 39, 40].

**Table 1:** General characteristics of included studies

First author	Year	Design	Type of critical condition	Delirium group					Non-delirium group					NOS score	NLR Cut-off point	SEN	SP	Delirium Definition
				N	NLR		PLR		N	NLR		PLR						
					Mean	SD	Mean	SD		Mean	SD	Mean	SD					
Guliyev,E	2016	P	-	30	29.22	5.20	101.29	11.01	34	29.42	5.13	102.71	12.22	9	-	-	-	DSM-IV
Egberts,A	2017	R	-	13	9.10	5.61	-	-	73	5.18	3.05	-	-	8	-	-	-	DSM-IV & V
Theologou,S	2018	P	POD	20	4.60	4.80	-	-	159	3.10	3.00	-	-	8	-	-	-	RASS and CAM-ICU
Kotfis,K.1	2019	P	PSD	121	6.71	9.65	-	-	639	4.55	5.51	-	-	7	-	-	-	CAM-ICU & DSM-V^a^
Kotfis,K.2	2019	P	PSD	172	4.74	4.05	-	-	829	3.43	2.25	-	-	8	4.86	42%	74%	CAM-ICU & DSM-V^a^
Kotfis,K.3	2019	R	POD	129	2.56	1.45	109.87	46.38	839	2.47	1.30	120.36	52.98	7	-	-	-	CAM-ICU & DSM-V^a^
He,R	2020	P	POD	182	4.67	1.62	-	-	598	2.68	1.26	-	-	6	3.5	75%	74%	CAM & DSM-IV
Ida,M	2020	R	POD	61	3.11	3.30	138.50	70.70	772	3.04	2.50	175.40	120.00	8	-	-	-	Validated chart-based method
Jiang,X	2020	R	-	29	17.10	12.70	306.50	240.50	290	13.60	10.90	202.20	144.90	7	-	-	-	CAM-ICU
Kalyoncuoglu,M	2020	R	POD	17	5.20	5.49	-	-	61	2.87	1.36	-	-	7	-	-	-	RASS & CAM-ICU
Yenibertiz,D	2020	R	-	126	15.86	12.89	345.86	211.93	86	10.10	11.23	235.70	209.08	8	-	-	-	DSM-V
Dardes,D	2021	P	PCD	14	9.37	8.91	-	-	42	6.02	5.14	-	-	7	-	-	-	4AT & CAM-ICU
Garcia-Grimshaw,M	2021	R	PCD	166	13.50	7.97	-	-	851	9.24	7.03	-	-	9	2.5	70%	52%	CAM-ICU & CAM brief version^b^
Guldolf,K	2021	R	PSD	201	3.96	2.76	-	-	313	2.66	1.78	-	-	8	-	-	-	DSM-V
Katipoglu,	2021	R	-	170	23.35	15.96	-	-	445	10.2	5.82	-	-	7	-	-	-	DSM
Kinoshita,H	2021	R	POD	20	4.16	1.94	-	-	77	1.94	0.77	-	-	8	-	-	-	ICDSC
Kotfis,K.4	2021	R	PCD	39	9.25	6.81	280.95	170.56	162	5.75	4.91	263.70	183.03	7	-	-	-	DSM V
Lechowicz,K	2021	R	POD	164	3.43	3.02	135.10	73.60	934	3.11	2.92	132.42	69.17	9	-	-	-	CAM-ICU & DSM-V^a^
Li,D	2021	R	-	393	15.23	12.46	-	-	864	17.45	12.98	-	-	8	-	-	-	CAM-ICU
Zhao, Y	2021	P	-	101					639					7	3.62	75%	63%	CAM
Oyama,T	2022	R	POD	20	3.34	1.42	171.66	62.23	90	2.23	1.31	161.33	61.02	8	2.45	85%	63%	ICDSC
Reznik,M.E	2021	R	PSD	157	9.00	10.40	-	-	127	6.40	5.50	-	-	7	-	-	-	DSM-V
Giorgi, D	2022	R	PCD	46	8.54	3.55	-	-	168	4.15	0.76	-	-	7	-	-	-	4AT& DSM
Li, J	2022	R	POD	48	5.62	3.89	-	-	48	3.98	1.98	-	-	7	-	-	-	DSM

*R* Retrospective, *P* prospective, *POD* Post-operative delirium, *PSD* Post-stroke delirium, *PCD* Post-COVID delirium, *N* Number, *SD* standard deviation, *NLR* Neutrophil to lymphocyte ratio, *PLR* Platelet to lymphocyte ratio, *NOS* Newcastle–Ottawa scale, *SEN* Sensitivity, *SP* Specificity

^a^CAM-ICU was used for screening, followed by DSM-V for confirmatory diagnosis

^b^All diagnoses confirmed by attending psychiatrist or neurologist

### Difference in NLR level between delirious and non-delirious patients

**Fig. 2:**
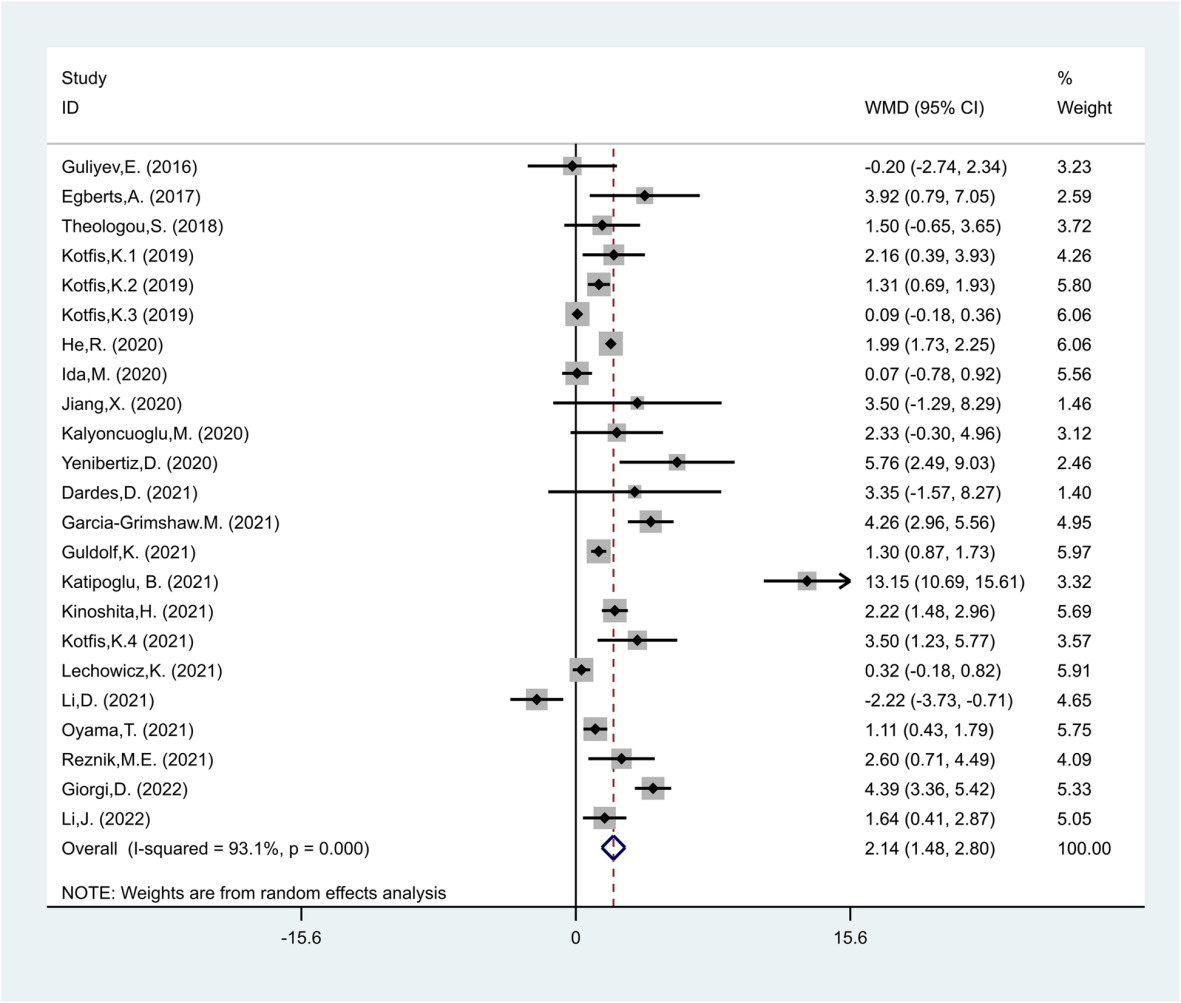
Meta-analysis of differences in NLR level between critically ill patients with and without delirium

NLR levels in the delirious group were compared with those of the non-delirious group in 23 cohort studies [17–35, 38–41] with 10,839 critically ill patients, of whom 2338 were diagnosed with delirium. Compared with the non-delirious group, the delirious group's NLR levels were significantly higher (WMD = 2.14; CI 95% = 1.48–2.80, *p* < 0.01). The included studies were statistically heterogeneous (I^2^ = 93.1%, *p* < 0.01); thus, the analysis used the random-effects model (Fig. 2).

**Fig. 3:**
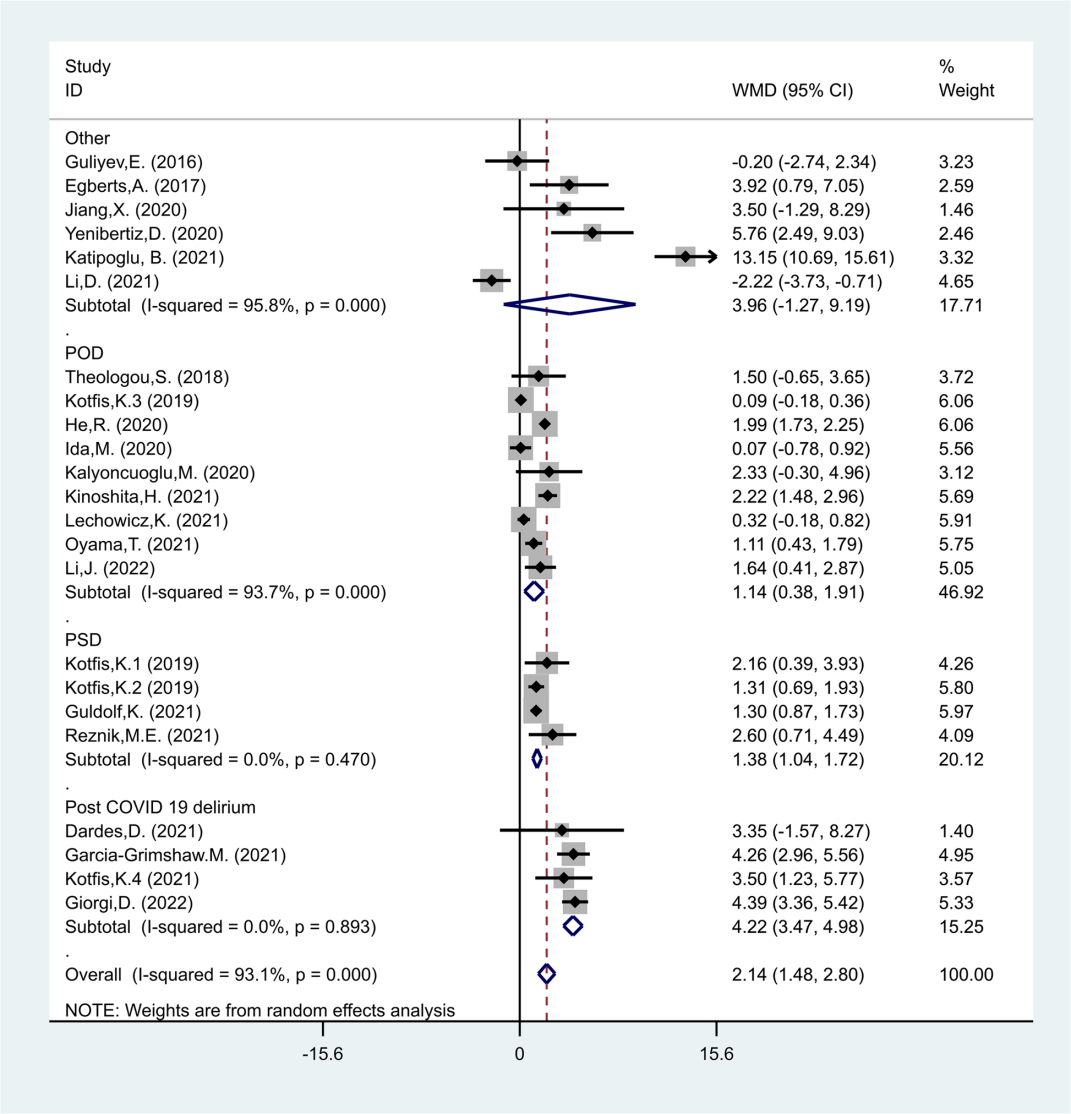
Subgroup analysis of differences in NLR level between critically ill patients with and without delirium according to disease type

In the subgroup analysis according to the type of critical condition, there were four studies on PCD [17, 19, 30, 38], including 1488 patients with COVID-19 of whom 265 developed delirium, and four studies on PSD [20, 27, 28, 33] including 2559 patients with stroke of whom 651 developed delirium, and nine studies on POD [21, 22, 25, 26, 29, 31, 34, 40, 41] including 4239 patients with stroke of whom 661 developed delirium. The NLR levels in patients of delirious group were significantly more than those of non-delirious group in studies on POD, PSD and PCD (WMD = 1.14, CI 95% = 0.38–1.91, *p* < 0.01, WMD = 1.38, CI 95% = 1.04–1.72, *p* < 0.001, and WMD = 4.22, CI 95% = 3.47–4.98, *p* < 0.001, respectively, Fig. 3).

**Fig. 4:**
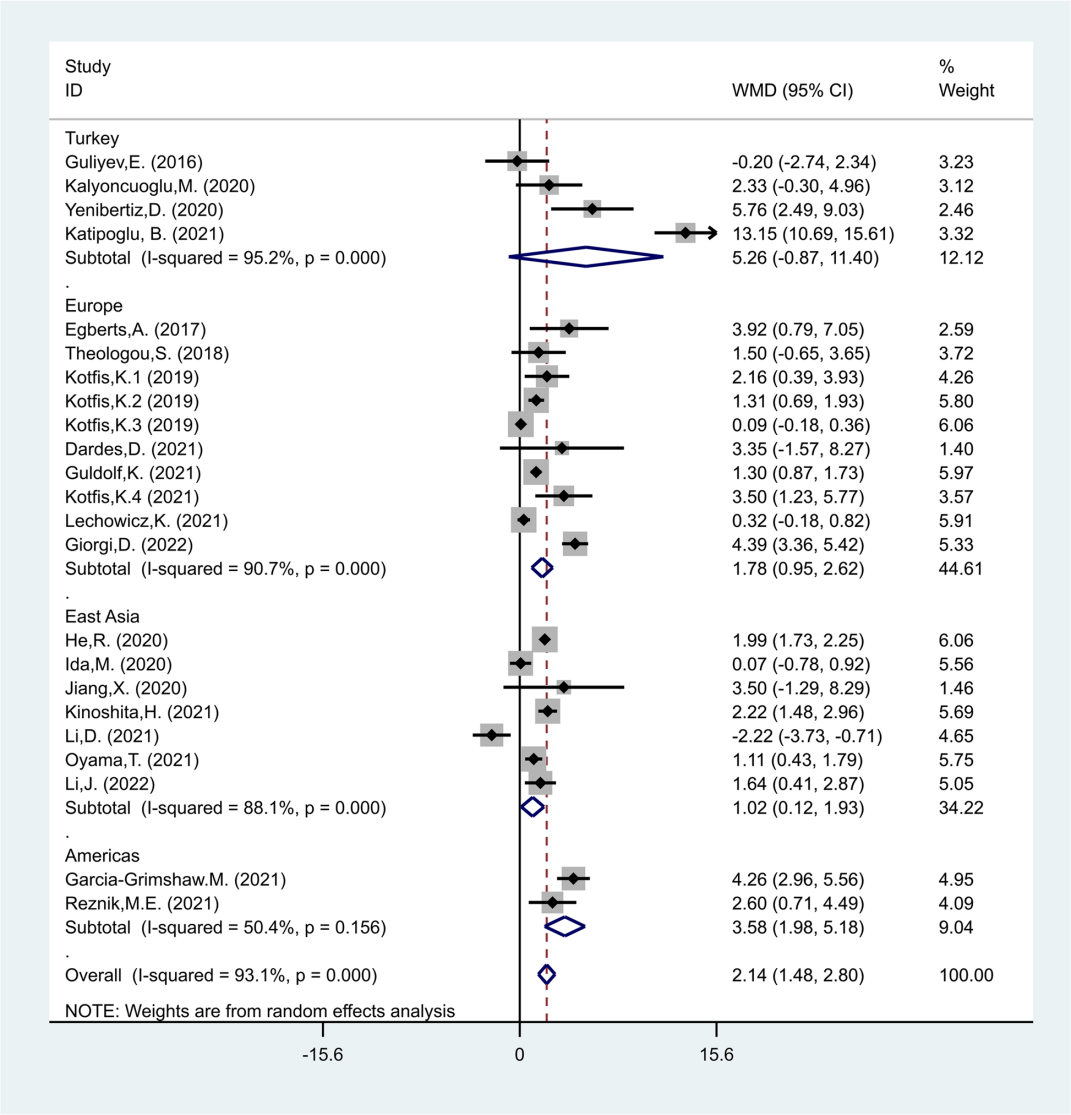
Subgroup analysis of differences in NLR level between critically ill patients with and without delirium according to study location

In another subgroup analysis according to the study location, there were four studies in Turkey [24, 25, 35, 39], including 969 patients of whom 343 developed delirium, ten studies in Europe [17, 18, 20, 27–31, 34, 38] including 5077 of whom 919 developed delirium, seven studies in East Asia [21–23, 26, 32, 40, 41] including 3492 patients of whom 753 developed delirium, and two studies in Americas [19, 33] including 1301 patients of whom 323 developed delirium. The NLR levels in patients of the delirious group were significantly more than those of the non-delirious group in studies in, Europe, East Asia and Americas (WMD = 1.87, CI 95% = 0.95–2.62, *p* < 0.001, and WMD = 1.02, CI 95% = 0.12–1.93, *p* = 0.02, and WMD = 3.58, CI 95% = 1.98–5.18, *p* < 0.001, respectively), but not in Turkey (WMD = 6.26, CI 95% = -0.87–11.40, *p* = 0.09, Fig. 4).

**Fig. 5:**
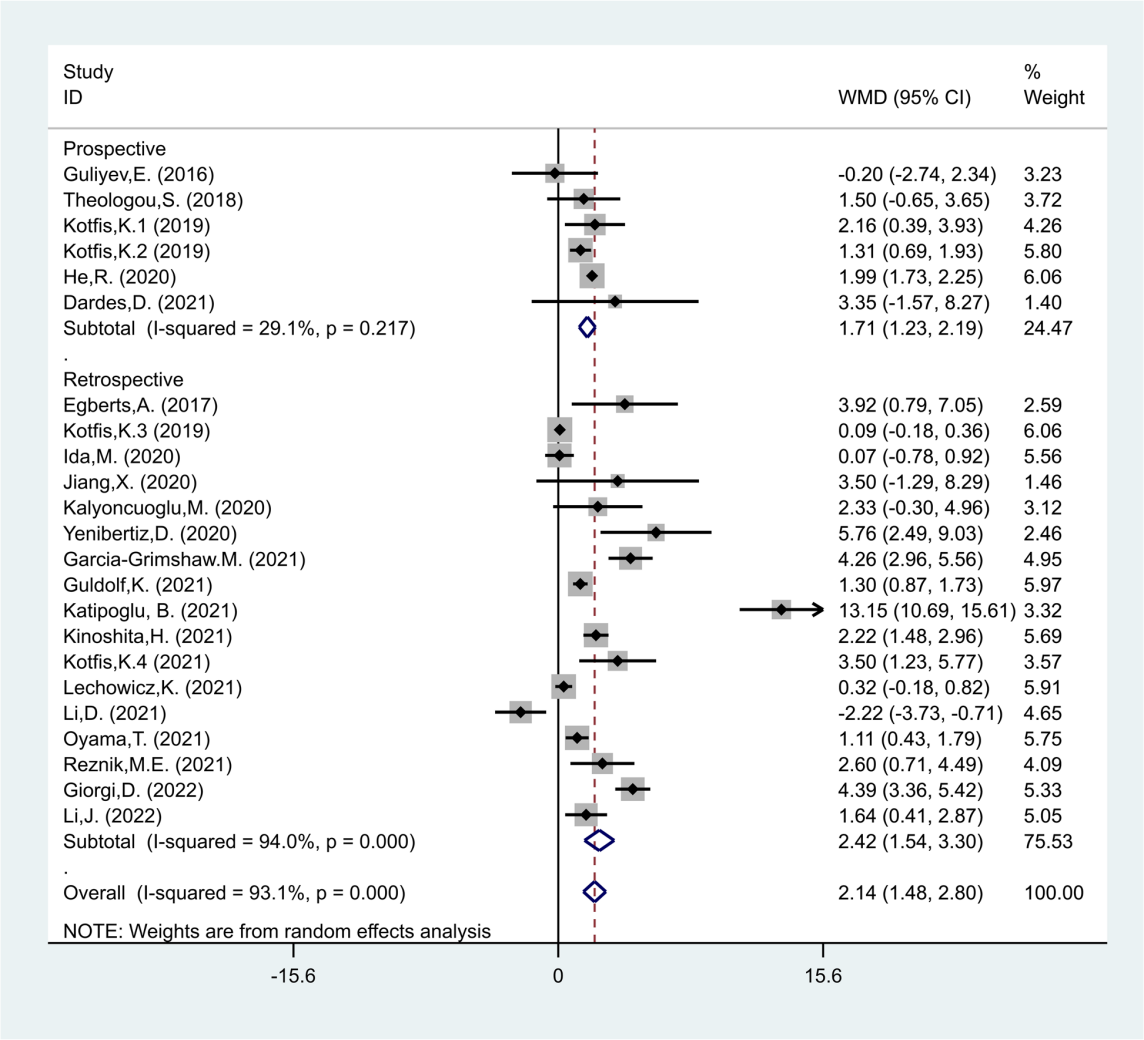
Subgroup analysis of differences in NLR level between critically ill patients with and without delirium according to study design

In the third subgroup analysis according to study design, there were 17 retrospective studies [18–20, 22, 23, 25, 26, 29–33, 35, 38–41], including 7990 patients of whom 1799 developed delirium and six prospective studies [17, 21, 24, 27, 28, 34] including 2840 patients of whom 539 developed delirium. The NLR levels in patients of the delirious group were significantly more than those of the non-delirious group in both prospective and retrospective studies (WMD = 1.71, CI 95% = 1.23–2.19, *p* < 0.001, and WMD = 2.42, CI 95% = 1.54–3.30, *p* < 0.001, respectively, Fig. 5).

**Fig. 6:**
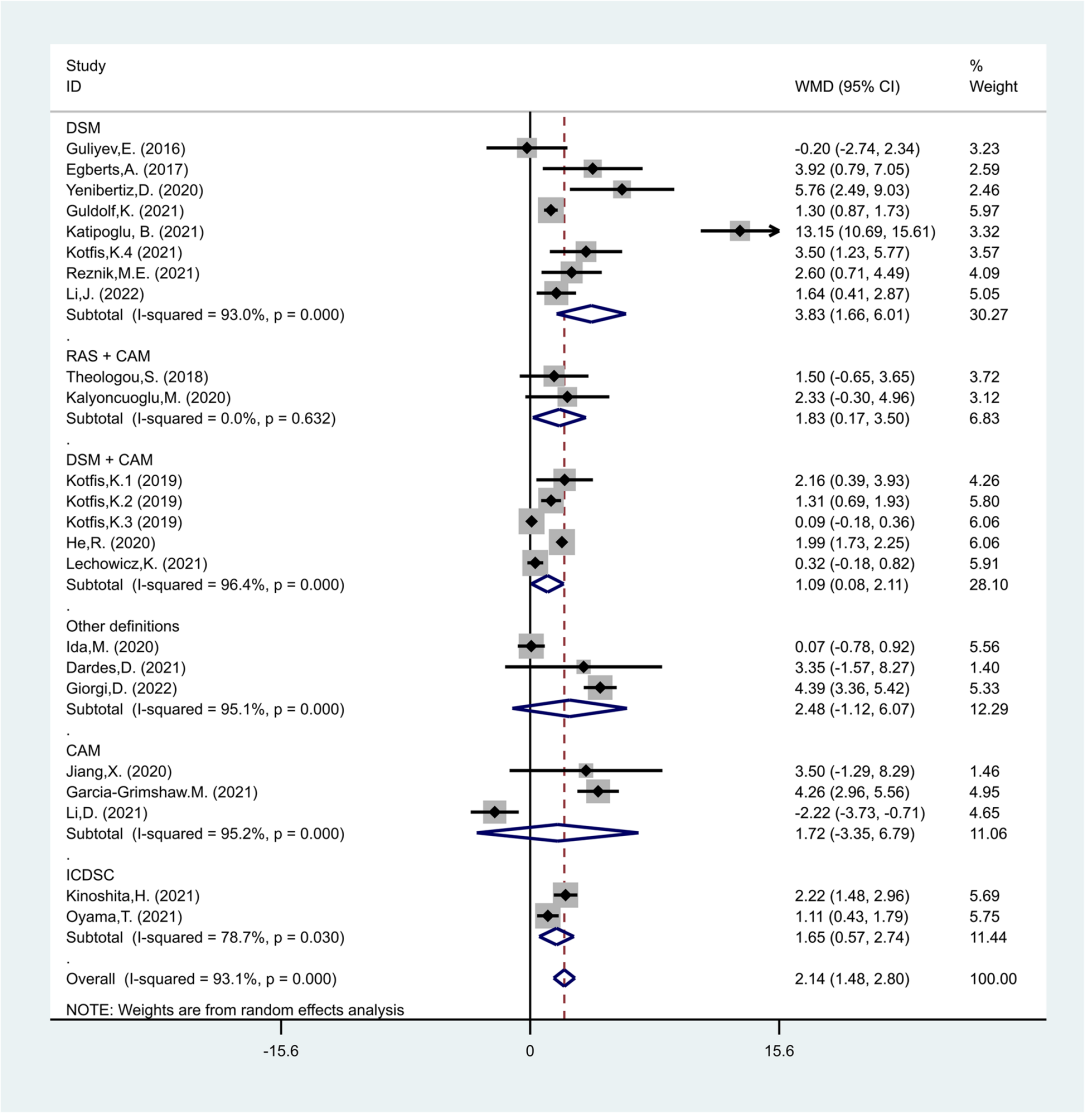
Subgroup analysis of differences in NLR level between critically ill patients with and without delirium according to definition of delirium

In the fourth subgroup analysis according to definition of delirium, there were eight studies which defined delirium according to DSM [30, 39, 40, 44–48], five studies according to DSM + CAM [31, 49–52], three studies according to CAM [53–55], two studies according to RAS + CAM [25, 56], two studies according to ICDSC [41, 57], one study according to 4AT + DSM [38], one study according to 4AT + CAM [17], and one study according to validated chart-based method [22]. The NLR levels in patients of the delirious group were significantly more than those of the non-delirious group in studies in which delirium was defined according to DSM (WMD = 3.83, CI 95% = 1.66–6.01, *p* = 0.001), RAS + CAM (WMD = 1.83, CI 95% = 0.17–3.50, *p* = 0.03), DSM + CAM (WMD = 1.09, CI 95% = 0.08–2.11, *p* = 0.03), and ICDSC (WMD = 1.65, CI 95% = 0.57–2.74, *p* = 0.003), but not in those in which delirium was defined according to CAM (WMD = 1.72, CI 95% = -3.35–6.79, *p* = 0.50, Fig. 6).

### Diagnostic value of NLR for differentiating between delirious and non-delirious patients

**Fig. 7:**
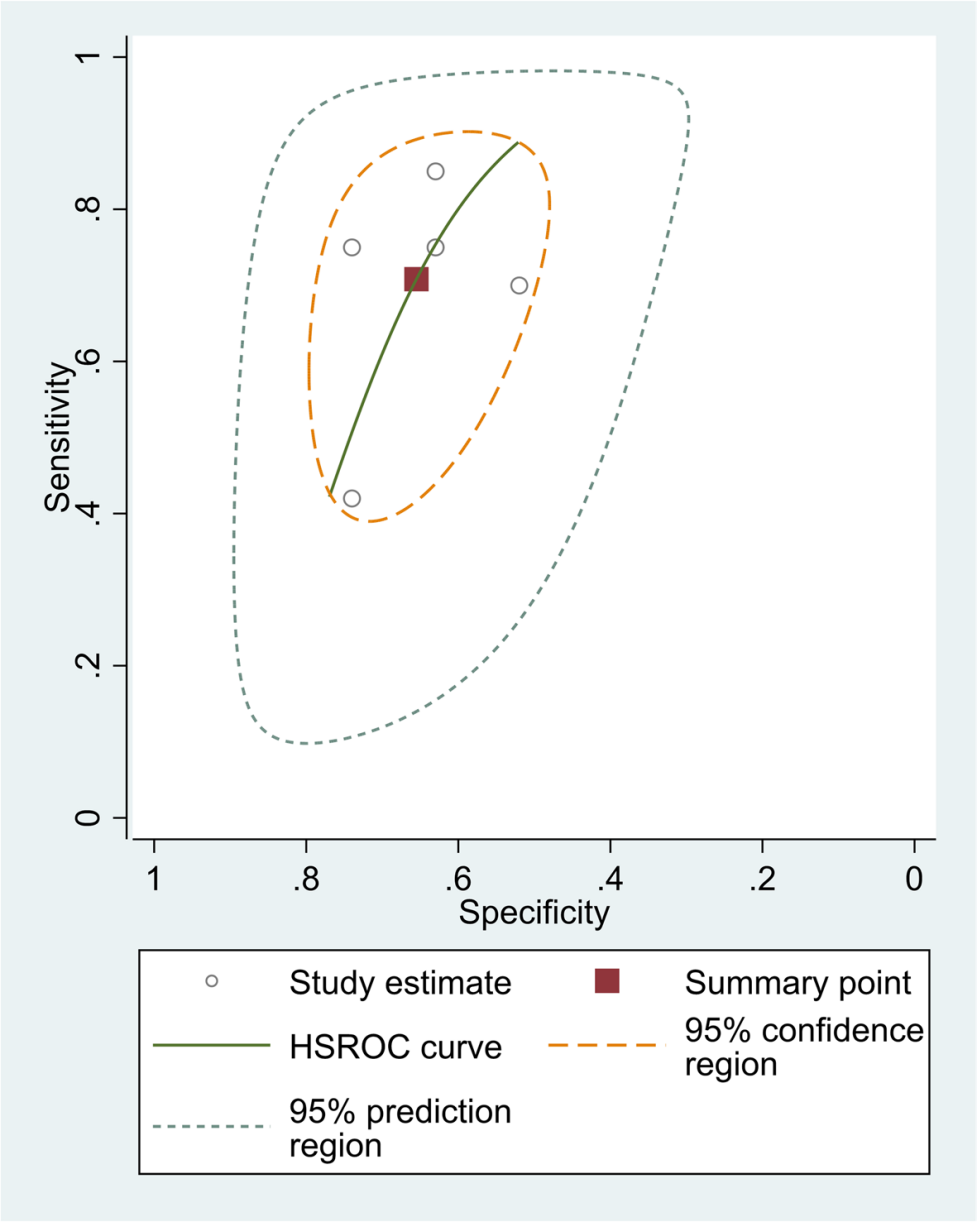
SROC curve of included studies

The pooled sensitivity of five studies was 70.80% (95% CI = 57.13%–81.51%), and the pooled specificity was 65.51% (95% CI = 57.87%–72.42%). The pooled positive likelihood ratio, negative likelihood ratio, and DOR of NLR were 2.05(95%CI = 1.65–2.54),0.44 (95%CI = 0.30–0.66), and 4.60(95%CI = 2.64–8.02), respectively (Fig. 7).

### Differences in PLR level between delirious and non-delirious patients

**Fig. 8:**
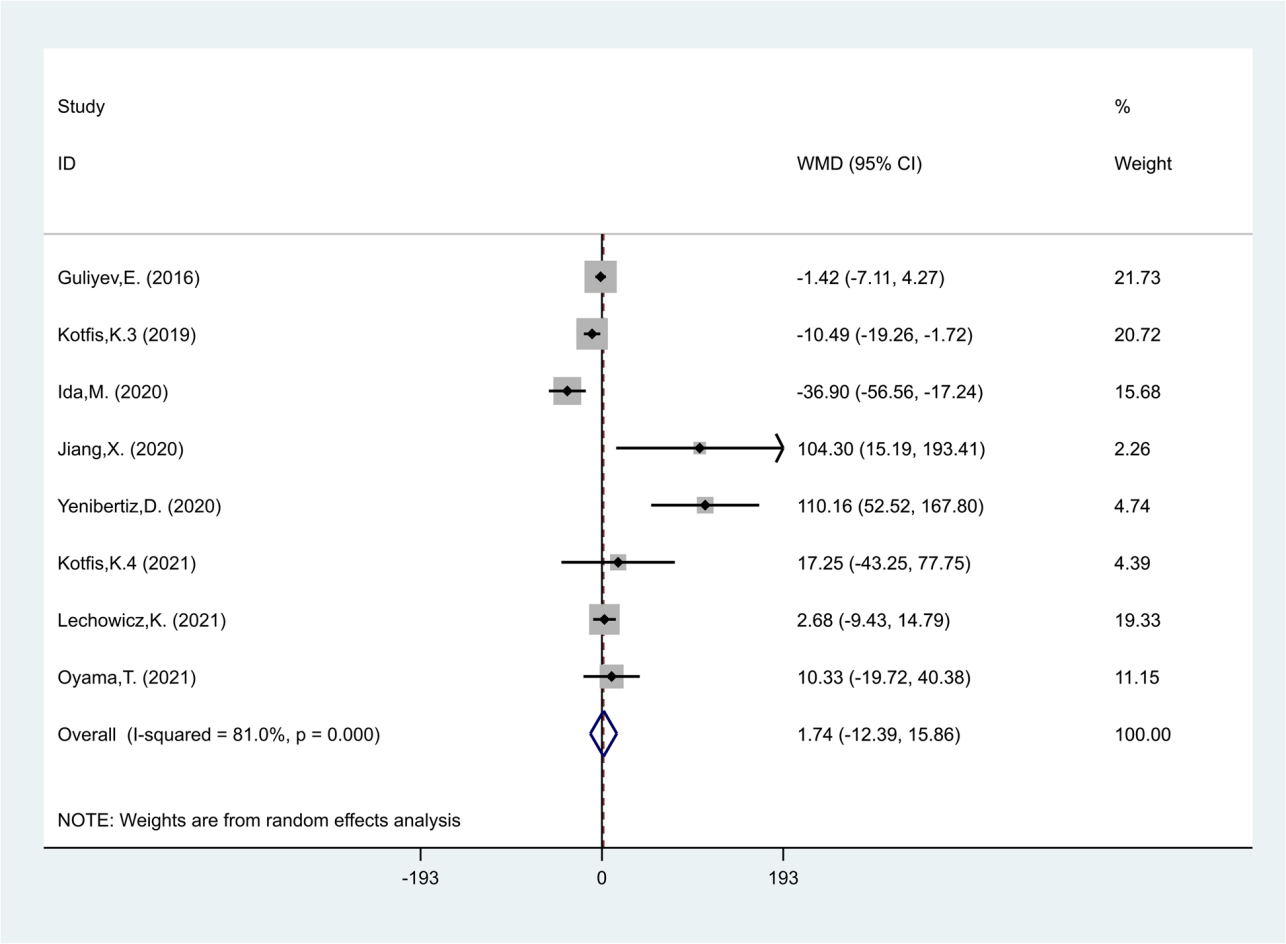
Meta-analysis of differences in PLR level between critically ill patients with and without delirium

PLR levels in the delirious group were compared with those of the non- delirious group in eight studies [22–24, 29–31, 35, 41] with 3805 critically ill patients, of which 598 patients were diagnosed with delirium, finally. Compared with the non-delirious group, the delirious group's PLR levels were not significantly different (WMD = 1.74; CI 95% = -12.39–15.86, *p* = 0.80). The included studies were statistically heterogeneous (I^2^ = 81.0%, *p* < 0.01); thus, the random-effects model was used for the meta-analysis (Fig. 8).

**Fig. 9:**
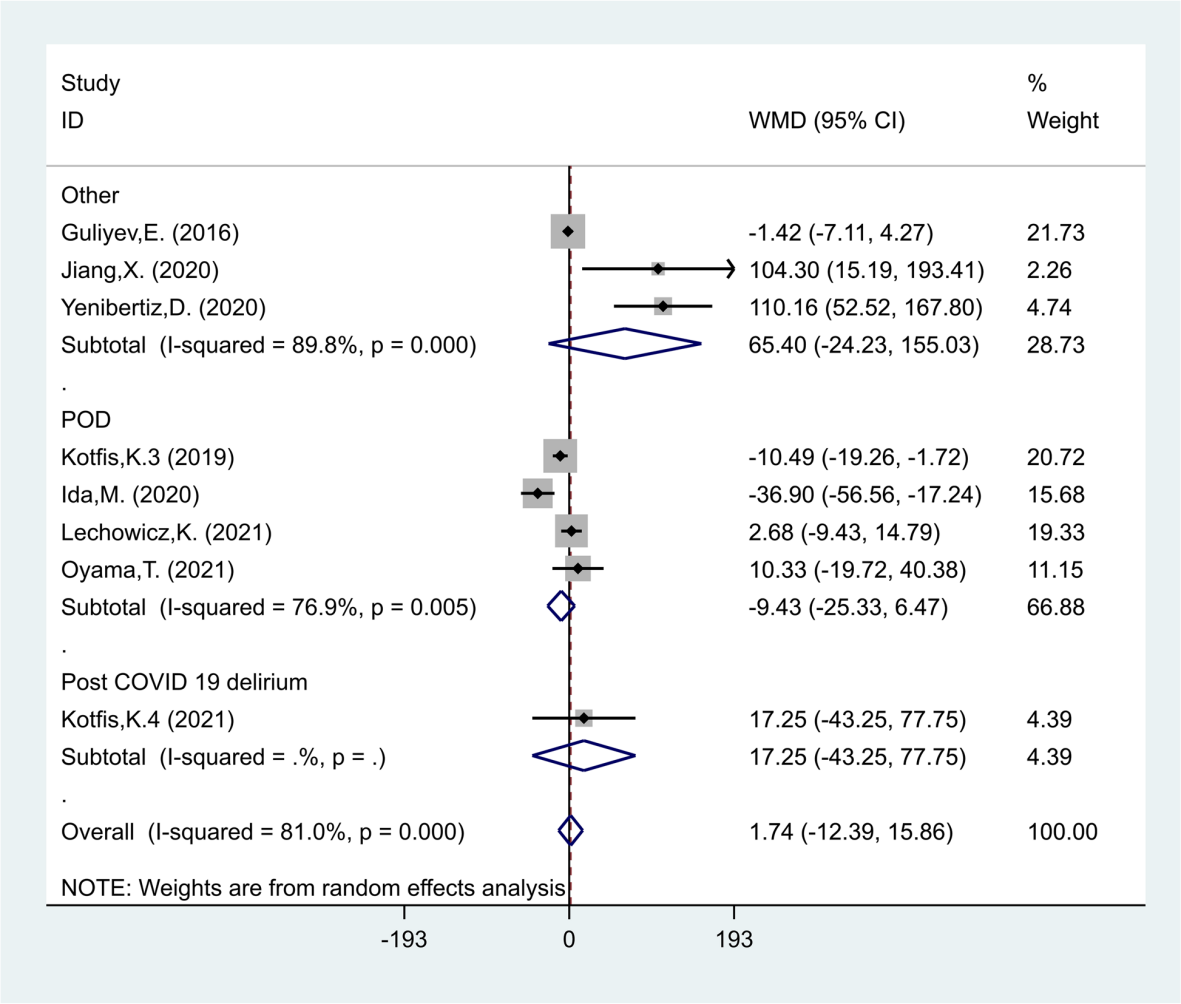
Subgroup analysis of differences in PLR level between critically ill patients with and without delirium according to disease type

In the subgroup analysis according to the type of critical condition, there were four studies on POD [22, 29, 31, 41], including 3009 patients, of whom 374 developed delirium. The PLR levels in patients of the delirious group were similar to those of the non- delirious group in patients undergoing surgery (WMD = -9.43, CI 95% = -25.33–6.47, *p* = 0.24). Also, one study [30], including 201 patients, of whom 39 developed delirium, reported that PLR were similar in the delirious and non- delirious groups in COVID-19 patients (WMD = 17.25, CI 95% = -43.25–77.75, *p* = 0.57, Fig. 9).

**Fig. 10:**
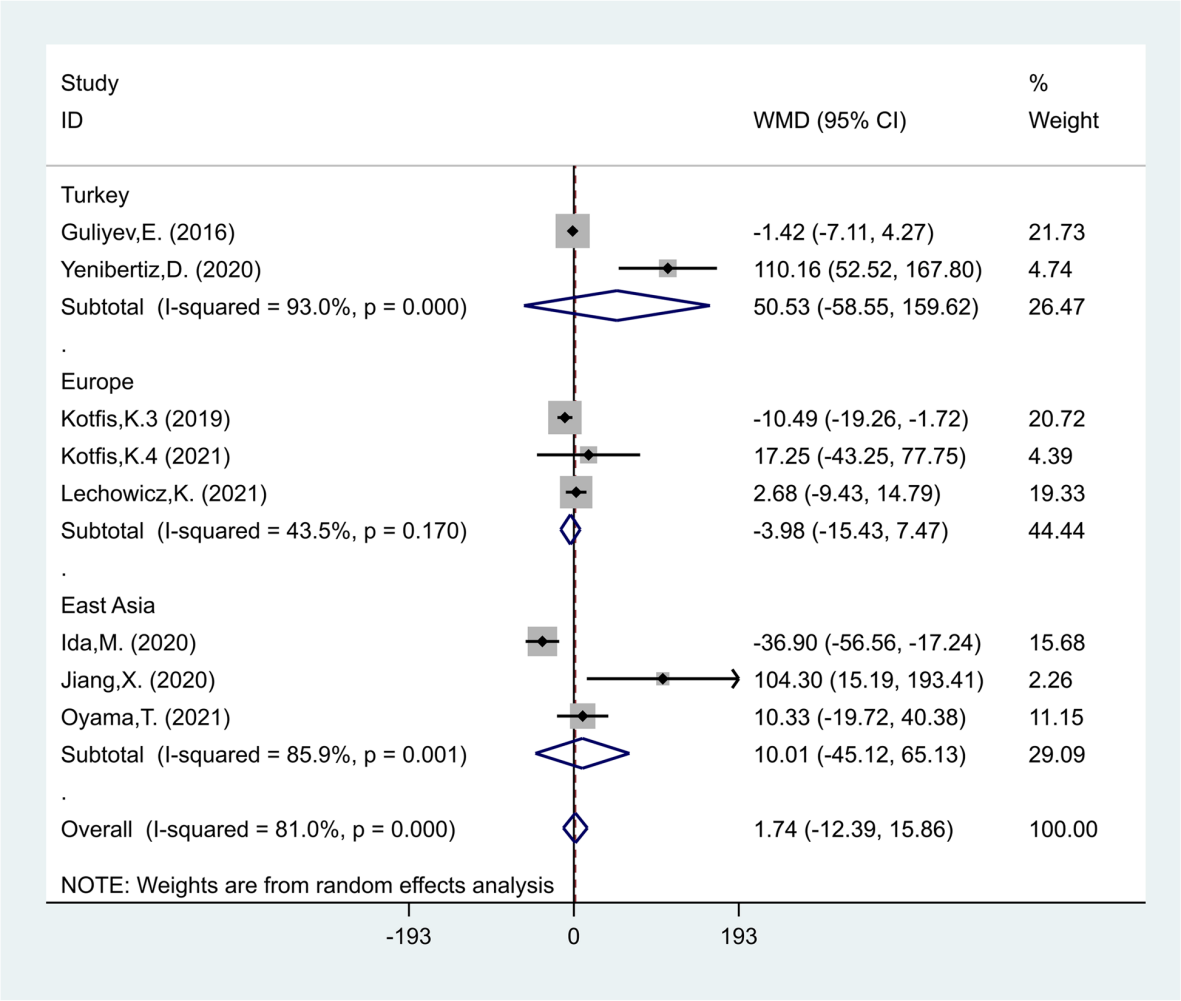
Subgroup analysis of differences in PLR level between critically ill patients with and without delirium according to study location

In another subgroup analysis according to the study location, there were two studies in Turkey [24, 35], including 156 patients of whom 120 developed delirium, three studies in Europe [29–31] including 2267 of whom 332 developed delirium, and three studies in East Asia [22, 23, 41] including 1262 patients of whom 110 developed delirium. The PLR levels in patients of the delirious group were similar to those of the non-delirious group in studies conducted in Turkey (WMD = 50.53, CI 95% = -58.55–159.62, *p* = 0.36), Europe (WMD = -3.98, CI 95% = -15.43–7.47, *p* = 0.49) and East Asia (WMD = 10.01, CI 95% = -45.12–65.13, *p* = 0.72, Fig. 10).

### Publication bias

**Fig. 11:**
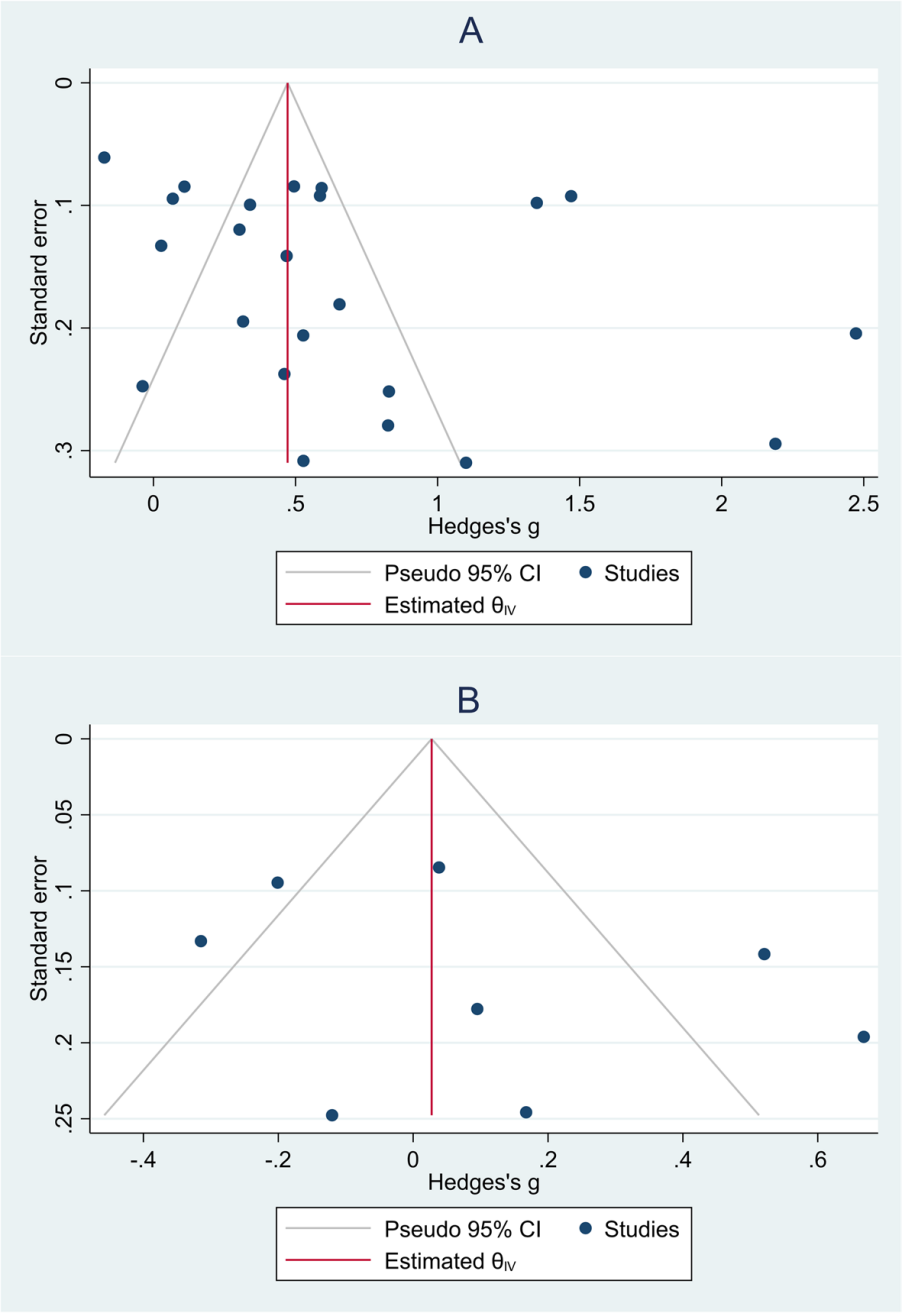
Funnel plots assessing the publication bias; **A** studies on NLR levels in critically ill patients; **B** studies on PLR levels in critically ill patients

The results of studies on the role of neither NLR [17–35, 38–41] nor PLR [22–24, 29–31, 35, 41] showed publication bias (Egger's test *p* = 0.10 and 0.48, respectively, Fig. 11).

## Discussion

The results of our study demonstrate that among critically ill patients, there was a significant difference in NLR values between those who developed delirium and those who did not. Clinically, this suggests that critically ill patients who develop delirium have higher NLR values and, thus, such a measure may have predictive utility in this setting.

Although heterogeneity exists in our results (I^2^ = 94.3%), most of our significance was not weakened by subgroup analysis when stratifying by study design, study location and type of critical condition. Interestingly, when stratifying for critical condition, PSD and PCD showed great precision with low and no heterogeneity, respectively. Such findings may indicate the NLR values are a more stable and reliable prognostic marker in PSD and PCD relative to POD. Furthermore, in subgroup analysis by geographic location, NLR proved to be a predictive marker in western countries, specifically in the Americas and Europe.

Despite significance in NLR values, we did not find similar significance in the analysis of PLR values – neither in the overall results nor in subgroup analysis. Ultimately, when accounting for geographic location and type of critical illness, our results suggest NLR is a unique inflammatory marker with potential to predict disease and aid clinical management.

### NLR, Neuroinflammation and the Neuroendocrine Axis

It has been postulated that elevated NLR values are secondary to a multifactorial process involving neuroendocrine and immunologic input [13]. Stress and severe illness can activate the hypothalamic–pituitary–adrenal (HPA) axis leading to elevations in cortisol that stimulate neutrophil de-margination and maturation, as well as lymphocyte apoptosis [14, 58–61]. Immunologically, severe illness increases the production of neutrophils from the bone marrow and can lead to lymphopenia via various proposed mechanisms [62–64]. Ultimately, a relative neutrophilia and lymphopenia can result, leading to an elevated NLR. Similarly, both stress and inflammatory stimuli can lead to increased platelet counts, which, in the setting of lymphopenia, would result in an elevated PLR [65, 66]. Although NLR and PLR are both markers of inflammation and stress, NLR may serve the unique utility of reflecting immune and neuroendocrine imbalances that can precipitate delirium.

Neutrophils are dominant players in innate immunity that serve to amplify pro-inflammatory responses [67]. On the other hand, lymphocytes are components of the adaptive immune system which serve to regulate immune responses [68]. In the setting of a high NLR, the pro-inflammatory activity of neutrophils may outweigh the regulatory function of lymphocytes, setting up a landscape for unregulated peripheral inflammation to transmit onto a vulnerable brain.

As it pertains to delirium, a neuroinflammatory hypothesis of pathogenesis has gained increasing attention [9, 10]. To date, substantial evidence supports the onset of neuroinflammation following systemic inflammatory processes [69]. Through transport across the blood–brain barrier (BBB), increased BBB permeability, afferent nerve stimulation, and transmission across circumventricular organs, it is hypothesized that a “cross-talk” exists between the peripheral immune system and the central nervous system [70–73]. Unregulated peripheral inflammation can take advantage of these pathways to induce neuroinflammatory processes that lead to altered neural networks that precipitate delirium.

Microglia activation is a strong marker of neuroinflammation and is thought to be important to delirium pathogenesis [74, 75]. Increased microglia activation is seen in post-mortem studies of patients with delirium, including those with PCD [76, 77]. Of interest, recent research has demonstrated the role of neutrophils in microglia activation. The cationic antimicrobial protein of 37kDA (CAP37), also known as heparin-binding protein, and IL-36ƴ are secreted by neutrophils and can stimulate microglia in vitro and in vivo, respectively [78, 79]. Moreover, research supports a prominent role for neutrophils in cytokine and chemokine secretion, a function once thought to be remote from the cell. Cytokines are classically elevated in patients with delirium and are thought to contribute to neuroinflammation through BBB disruption and glial cell activation [11, 80].

Inflammatory mediators such as IL-1B, IL-6, IL-8, IL-10, TNF-alpha, and C-reactive protein (CRP), have all shown associations with delirium [11]. Currently, one of the most well-studied cytokines in delirium is IL-6. Numerous studies have demonstrated its elevation in delirious patients, and such results have been substantiated in a recent meta-analysis focused on POD [11, 81–83]. Further, in pre-clinical models of delirium, targeting cytokines, such as IL-6, have shown therapeutic promise [68, 72–74]. Altogether, such evidence suggests a role for systemic inflammation in the onset of delirium and such processes may be reflected in elevated NLR values.

An aberrant stress response within the neuroendocrine axis has also been proposed to lead to elevated NLR measures in patients with delirium [75]. As previously described, glucocorticoids released from the HPA axis can result in hematopoietic changes that lead to elevated NLRs. However, within the central nervous system, cortisol can also directly impact neuroregulatory processes leading to neuroinflammation, neuronal dysregulation, and oxidative stress [84]. In fact, critically ill patients are known to undergo dynamic neuroendocrine stress responses, and such responses have been demonstrated as poor prognostic indicators of disease. (Van den Berghe 2002).

Several studies have confirmed elevations of serum cortisol in patients with delirium, including POD, sepsis-associated delirium, and PSD [11, 85–87]. Further, among older adults, dexamethasone non-suppression is associated with a greater risk for delirium [88, 89]. Although cortisol is historically known for its immunosuppressive and regulatory functions, it is hypothesized that chronic elevations or a dysregulation of the HPA axis can lead to neurologic insults that precipitate delirium [90, 91].

Ultimately, based on our results and a growing understanding of delirium pathogenesis, NLR appears to be a unique inflammatory marker that reflects important pathophysiologic cascades which take place in delirium patients. Our ability to understand these dynamic biological processes may help us to better treat disease, as well as best integrate new prognostic measures into a clinical setting.

### Clinical implications

Currently, delirium remains a difficult condition to predict, diagnose and treat [1, 92, 93]. It is proposed that approximately 30–40% of delirium can be prevented [94, 95]. Given that only one episode of delirium is needed to impact long-term morbidity and mortality, it is essential to develop advanced, objective measures to better prevent and diagnose disease [8, 96]. To date, a number of inflammatory markers have been studied for this purpose. Most notably, IL-6, CRP, cortisol, IL-8, and S100-beta have shown promise [11]. However, inconsistent results and inherent limitations, such as cost and resource burden, have hampered their implementation [12].

NLR is a measure that is readily obtained on admission from a white blood cell differential and is associated with no additional cost or labor. Currently, a few studies show that NLR can predict delirium with relatively high sensitivity and specificity [97, 98]. However, associated sensitivity and specificity vary across studies and overall strength is inconsistent. Ultimately, NLR as an isolated measure is unlikely to reliably predict delirium. As such, NLR may be best employed as an adjunctive measure with additional prognostic markers. For example, in a prospective study of PSD, a prediction model including NLR, NIHSS score, leukocyte count, CRP and measures of neurologic dysfunction, yielded an area under the receiver operator curve of 0.801 with associated sensitivity and specificity of 0.813 and 0.673, respectively [28]. Additionally, in a study by Kinoshita et al. evaluating POD, the addition of NLR to a pre-validated prediction model for delirium in ICU patients raised the area under the receiver operative curve to from 0.60 to 0.87 [26]. Additional studies included in this meta-analysis have shown similar levels of strength with prediction models incorporating NLR [20, 22, 29]. Altogether, the use of NLR in conjunction with additional predictive measures holds potential to reliably predict delirium in critically ill patients. As increasing research is conducted on this matter, future meta-analyses should be completed to validate prediction models.

Furthermore, validated cut-off values for NLR may prove more practical in a clinical setting. However, in this context, it is important to consider that the degree to which NLR rises from healthy baseline varies according to the type of critical condition [13]. For example, NLR values are characteristically higher in patients with severe COVID-19 pneumonia as compared to those with acute ischemic stroke [13]. Due to variations in NLR based on critical condition, and the resulting small sample sizes when stratifying for critical condition in our meta-analysis, we did not analyze the pooled results to determine a cut-off value. However, among the studies in our meta-analysis, a few authors did report optimal cut-off values. Specifically, optimal cut-off values were reported as 3.5 for post-operative orthopedic patients, 2.45 for patients undergoing esophagectomy, 3.62 for older internal medicine patients, 4.86 in post-stroke patients, and 2.5 in COVID-19 patients [21, 26–28]. With further research and additional prospective studies, it should be possible to validate optimal cut-off values to predict delirium based on type of critical condition.

### Limitations, strengths and future directions

Our study has a few limitations that are important to address. Foremost, our study revealed high heterogeneity for the overall pooled results of studies evaluating NLR ( I^2^ = 94.3%), thus limiting wider generalizations. To further explore heterogeneity, subgroup analysis was completed. We speculated that study design, definition of delirium, type of critical illness, and study location could be potential confounders and sources of heterogeneity.

Interestingly, on subgroup analysis for type of critical illness, POD demonstrated substantially greater heterogeneity (I^2^ = 97%) as compared to PSD and PCD (I^2^ = 42.2% and I^2^ = 0.0%, respectively). The relatively increased heterogeneity in these studies could be due to a variety factors. One such explanation may be inherent variations in inflammatory and neuroendocrine responses that take place with different surgical interventions. A variety of post-operative patients were included in this meta-analysis, including cardiac, orthopedic, and gastrointestinal patients. The predictive utility of NLR in POD may vary according to procedure performed, thus, contributing to the variation. As more research is conducted, future meta-analyses dedicated to the study of POD which sub-analyze based on procedure type would be warranted to better answer this question. In addition, the timing of laboratory sample collection varied between studies and one study in this subgroup demonstrated a much larger effect size compared to the others. All such factors may have skewed the results and contributed to heterogeneity.

Despite aforementioned findings, none of the subgroup analyses could entirely explain the heterogeneity. Thus, we speculated on additional sources of heterogeneity. As previously referenced, another possible source of heterogeneity could be the timing in which hematologic laboratory values were obtained. All studies did not specify timing of collection and, among those which did, timing appeared to vary. As such, future studies analyzing the prognostic value of NLR may benefit from standardized protocols that specify timing of blood sample collection, as well as diagnostic tools.

Moreover, on completion of subgroup analysis by geographic location, there was a loss of effect size for studies completed in East Asia and Turkey. We speculated two possible explanations for these findings. First, studies have demonstrated that baseline NLR values can vary based on race and geographic location [13]. As such, there may also be an inherent genetic difference in the way the immune system responds to pathologic stress. Thus, it is possible that certain populations may not experience characteristic alterations in hematopoiesis following critical illness. Second, there may also be variation in study methodology according to geographic region—for example, divergence in tools used to detect delirium and to train individuals to do so.

Finally, the study sample size measuring PLR values was small and, thus, our results may not be sufficiently powered to make a conclusion regarding such values. Additional studies will be warranted to confidently assess PLR’s association with delirium in a future meta-analysis with a larger sample size.

Despite limitations, our study also has a number of strengths. Foremost, this systematic review incorporated an extensive search of the literature according to a standardized protocol and was augmented by manual search to ensure thorough review. In addition, there was no publication bias detected in this meta-analysis, thus strengthening validity of results. Finally, study quality, as assessed by NOS, was ≥ 7 for all studies, which likewise further strengthens the validity of our results.

## Conclusion

In conclusion, delirium remains a condition that is difficult to prevent, diagnose and treat. As a result, there has been growing interest in developing better predictive markers for disease. Overall, the results of our study support the predictive utility of NLR in the development of delirium among critically ill patients. Clinically, NLR may be best utilized in conjunction with additional prognostic markers to achieve robust predictive strength. However, based on our results, further studies are warranted to better clarify the prognostic utility of NLR in Eastern countries and in POD. Ultimately, our findings support NLR to be a promising prognostic measure that, with further validation, can be readily integrated into clinical settings to aid in the prediction and prevention of delirium.

## Supplementary Information

**Additional file 1.**

**Additional file 2.**

**Additional file 3.**

## Data Availability

The dataset supporting the conclusions of this article is included within the article.

## Abbreviations

NLR: Neutrophil to lymphocyte ratio
PLR: Platelet to lymphocyte ratio
ICU: Intensive care unit
WMD: Weighted mean difference
95% CI: 95% Confidence interval
POD: Post-operative delirium
PCD: Post-COVID delirium
PSD: Post-stroke delirium
CAP37: Cationic antimicrobial protein of 37kDA
CRP: C-reactive protein
