# Supplementary material for: Neutrophil to lymphocyte ratio and platelet to lymphocyte ratio as prognostic predictors for delirium in critically ill patients: a systematic review and meta-analysis
Source: BMC Anesthesiol. 2023 Feb 21;23:58. doi: 10.1186/s12871-023-01997-2 (PMC9942068; doi:10.1186/s12871-023-01997-2)
Supplement: Supplementary file 2 — Additional file 2. [file 12871_2023_1997_MOESM2_ESM.docx]

**Search strategy**

**Title:** Neutrophil to lymphocyte ratio and platelet to lymphocyte ratio as prognostic predictors for delirium in critically ill patients: a systematic review and meta-analysis

**Scopus**

N=420

ALL ( ( neutrophil AND lymphocyte AND ratio ) OR ( neutrophil-to-lymphocyte ) OR nlr OR ( platelet AND lymphocyte AND ratio ) OR ( platelet-to-lymphocyte ) OR plr ) AND ALL ( delirium ) )

**Web of Science**

N=48

(Neutrophil AND lymphocyte AND ratio) OR Neutrophil-to- lymphocyte OR NLR OR (Platelet AND lymphocyte AND ratio) OR platelet- to- lymphocyte OR PLR (All Fields) and Delirium (All Fields)

**PubMed**

N=40

(("Neutrophil"[All Fields] AND "lymphocyte"[All Fields] AND "ratio"[All Fields]) OR "Neutrophil-to-lymphocyte"[All Fields] OR "NLR"[All Fields] OR ("Platelet"[All Fields] AND "lymphocyte"[All Fields] AND "ratio"[All Fields]) OR "platelet-to-lymphocyte"[All Fields] OR "PLR"[All Fields]) AND ("delirium"[MeSH Terms] OR "delirium"[All Fields] OR "deliriums"[All Fields])
