# Supplementary material for: Neutrophil to lymphocyte ratio and platelet to lymphocyte ratio as prognostic predictors for delirium in critically ill patients: a systematic review and meta-analysis
Source: BMC Anesthesiol. 2023 Feb 21;23:58. doi: 10.1186/s12871-023-01997-2 (PMC9942068; doi:10.1186/s12871-023-01997-2)
Supplement: Supplementary file 3 — Additional file 3. [file 12871_2023_1997_MOESM3_ESM.docx]

| NOS items | | Kotfis,K.4 | Egberts,A. | Kinoshita,H. | Kotfis,K.1 | Theologou,S. | He,R. | Guliyev,E. | Kotfis,K.3 | Yenibertiz,D | Garcia-Grimshaw,M. | Kotfis,K.2 | Zhao,Y. | Ida,M. | Jiang,X. | Guldolf,K. | Kalyoncuoglu,M. | Dardes,D. | Katipoglu, | Lechowicz,K. | Li,D. | Oyama,T. | Reznik,M.E. | Giorgi, D. | Li, J. |
| --- | --- | --- | --- | --- | --- | --- | --- | --- | --- | --- | --- | --- | --- | --- | --- | --- | --- | --- | --- | --- | --- | --- | --- | --- | --- |
| Selection | Is the case definition adequate? | ● | ⁎ | ● | ⁎ | ⁎ | ● | ⁎ | ⁎ | ⁎ | ⁎ | ⁎ | ⁎ | ⁎ | ● | ⁎ | ● | ⁎ | ⁎ | ⁎ | ⁎ | ⁎ | ⁎ | ● | ● |
|  | Representativeness of the cases | ⁎ | ⁎ | ⁎ | ⁎ | ⁎ | ● | ⁎ | ⁎ | ● | ⁎ | ⁎ | ● | ⁎ | ⁎ | ⁎ | ⁎ | ● | ● | ⁎ | ⁎ | ⁎ | ● | ⁎ | ⁎ |
|  | Selection of Controls | ⁎ | ⁎ | ⁎ | ⁎ | ⁎ | ⁎ | ⁎ | ⁎ | ⁎ | ⁎ | ⁎ | ⁎ | ⁎ | ⁎ | ⁎ | ⁎ | ⁎ | ⁎ | ⁎ | ⁎ | ⁎ | ⁎ | ⁎ | ⁎ |
|  | Definition of Controls | ⁎ | ⁎ | ⁎ | ● | ⁎ | ⁎ | ⁎ | ⁎ | ⁎ | ⁎ | ⁎ | ⁎ | ⁎ | ⁎ | ⁎ | ⁎ | ⁎ | ⁎ | ⁎ | ⁎ | ⁎ | ⁎ | ⁎ | ⁎ |
| Comparability | Study controls  for the most  important factor | ⁎ | ⁎ | ⁎ | ⁎ | ⁎ | ● | ⁎ | ● | ⁎ | ⁎ | ⁎ | ⁎ | ⁎ | ⁎ | ⁎ | ⁎ | ● | ⁎ | ⁎ | ⁎ | ⁎ | ⁎ | ⁎ | ⁎ |
|  | Study controls for the second  important factor | ⁎ | ⁎ | ⁎ | ⁎ | ⁎ | ⁎ | ⁎ | ⁎ | ⁎ | ⁎ | ⁎ | ⁎ | ⁎ | ⁎ | ⁎ | ● | ⁎ | ⁎ | ⁎ | ⁎ | ⁎ | ⁎ | ⁎ | ⁎ |
| Exposure | Was the measurement  method of NLR described? | ● | ● | ⁎ | ● | ● | ⁎ | ⁎ | ● | ⁎ | ⁎ | ● | ● | ● | ● | ● | ⁎ | ⁎ | ● | ⁎ | ● | ● | ● | ● | ● |
|  | Were the methods of  measurements  similar for cases  and controls? | ⁎ | ⁎ | ⁎ | ⁎ | ⁎ | ⁎ | ⁎ | ⁎ | ⁎ | ⁎ | ⁎ | ⁎ | ⁎ | ⁎ | ⁎ | ⁎ | ⁎ | ⁎ | ⁎ | ⁎ | ⁎ | ⁎ | ⁎ | ⁎ |
|  | Non-response rate | ⁎ | ⁎ | ⁎ | ⁎ | ⁎ | ⁎ | ⁎ | ⁎ | ⁎ | ⁎ | ⁎ | ⁎ | ⁎ | ⁎ | ⁎ | ⁎ | ⁎ | ⁎ | ⁎ | ⁎ | ⁎ | ⁎ | ⁎ | ⁎ |
|  | Total Score | 7 | 8 | 8 | 7 | 8 | 6 | 9 | 7 | 8 | 9 | 8 | 7 | 8 | 7 | 8 | 7 | 7 | 7 | 9 | 8 | 8 | 7 | 7 | 7 |

Methodological quality assessment using the Newcastle–Ottawa scale (NOS)

**Title:** Neutrophil to lymphocyte ratio and platelet to lymphocyte ratio as prognostic predictors for delirium in critically ill patients: a systematic review and meta-analysis
